# RadImageNet: An Open Radiologic Deep Learning Research Dataset for Effective Transfer Learning

**DOI:** 10.1148/ryai.210315

**Published:** 2022-07-27

**Authors:** Xueyan Mei, Zelong Liu, Philip M. Robson, Brett Marinelli, Mingqian Huang, Amish Doshi, Adam Jacobi, Chendi Cao, Katherine E. Link, Thomas Yang, Ying Wang, Hayit Greenspan, Timothy Deyer, Zahi A. Fayad, Yang Yang

**Affiliations:** From the BioMedical Engineering and Imaging Institute (X.M., Z.L., P.M.R., C.C., K.E.L., T.Y., H.G., Z.A.F., Y.Y.) and Department of Diagnostic, Interventional and Molecular Radiology (P.M.R., B.M., M.H., A.D., A.J., Z.A.F., Y.Y.), Icahn School of Medicine at Mount Sinai, Leon and Norma Hess Center for Science and Medicine, 1470 Madison Ave, New York, NY 10029; Department of Mathematics, University of Oklahoma, Norman, Okla (Y.W.); Department of Radiology, Cornell Medicine, New York, NY (T.D.); and Department of Radiology, East River Medical Imaging, New York, NY (T.D.).

**Keywords:** CT, MR Imaging, US, Head/Neck, Thorax, Brain/Brain Stem, Evidence-based Medicine, Computer Applications–General (Informatics)

## Abstract

**Purpose:**

To demonstrate the value of pretraining with millions of radiologic images compared with ImageNet photographic images on downstream medical applications when using transfer learning.

**Materials and Methods:**

This retrospective study included patients who underwent a radiologic study between 2005 and 2020 at an outpatient imaging facility. Key images and associated labels from the studies were retrospectively extracted from the original study interpretation. These images were used for RadImageNet model training with random weight initiation. The RadImageNet models were compared with ImageNet models using the area under the receiver operating characteristic curve (AUC) for eight classification tasks and using Dice scores for two segmentation problems.

**Results:**

The RadImageNet database consists of 1.35 million annotated medical images in 131 872 patients who underwent CT, MRI, and US for musculoskeletal, neurologic, oncologic, gastrointestinal, endocrine, abdominal, and pulmonary pathologic conditions. For transfer learning tasks on small datasets—thyroid nodules (US), breast masses (US), anterior cruciate ligament injuries (MRI), and meniscal tears (MRI)—the RadImageNet models demonstrated a significant advantage (*P* < .001) to ImageNet models (9.4%, 4.0%, 4.8%, and 4.5% AUC improvements, respectively). For larger datasets—pneumonia (chest radiography), COVID-19 (CT), SARS-CoV-2 (CT), and intracranial hemorrhage (CT)—the RadImageNet models also illustrated improved AUC (*P* < .001) by 1.9%, 6.1%, 1.7%, and 0.9%, respectively. Additionally, lesion localizations of the RadImageNet models were improved by 64.6% and 16.4% on thyroid and breast US datasets, respectively.

**Conclusion:**

RadImageNet pretrained models demonstrated better interpretability compared with ImageNet models, especially for smaller radiologic datasets.

**Keywords:** CT, MR Imaging, US, Head/Neck, Thorax, Brain/Brain Stem, Evidence-based Medicine, Computer Applications–General (Informatics)

*Supplemental material is available for this article.*

Published under a CC BY 4.0 license.

See also the commentary by Cadrin-Chênevert in this issue.

## Graphical abstract

**Figure:**
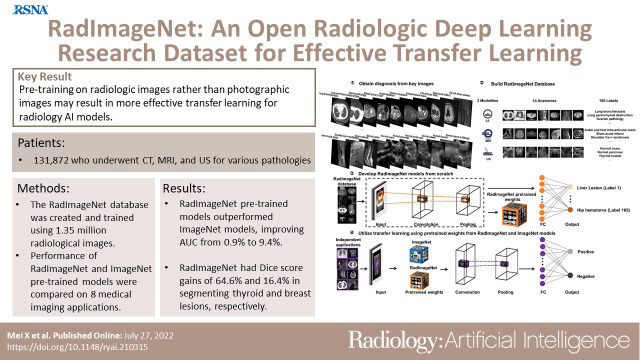

**Summary**

RadImageNet pretrained models could be an effective starting point for transfer learning in radiologic imaging artificial intelligence applications.

**Key Points**

■ The RadImageNet database is a large-scale dataset consisting of 1.35 million radiologic images covering CT, MRI, and US modalities and 11 anatomic regions, which were annotated by fellowship-trained and board-certified radiologists.
■ RadImageNet pretrained models demonstrated superior performance in the classification of eight independent medical applications as compared with ImageNet pretrained models, showing improvements from 0.9% to 9.4% for area under the receiver operating characteristic curve.
■ RadImageNet pretrained models were also able to interpret results more consistently compared with ImageNet pretrained models in thyroid and breast applications, demonstrating Dice score gains of 64.6% and 16.4% in segmenting the lesions, respectively.

## Introduction

ImageNet (1,2) is a dataset composed of millions of images of the natural world. ImageNet, as an open source dataset, has been a central resource for deriving sophisticated models in computer vision. Unlike ImageNet, publicly available medical imaging databases for research purposes are in scarcity because of the difficulty of curation, anonymization, or annotations of clinical data (3). Only a few well-curated annotated medical imaging datasets with high-quality ground truth pathologic labels are publicly available. The U.K. Biobank (4,5) contains multimodal images in more than 100 000 participants. However, most U.K. Biobank participants are healthy. The Cancer Imaging Archive (6) contains a large database of national lung screening trials, which consists of low-dose helical CT images in 53 454 participants (7). The National Institutes of Health ChestX-ray8 contains 108 948 chest radiographs in 32 717 patients, with eight disease labels (8). Most current publicly available medical imaging datasets are limited in sample size, diversity of disease labels, or modality variety for artificial intelligence (AI) practice or lack pathologic findings.

Limited sample size may create barriers to developing successful AI models. In cases of limited sample size, transfer learning (9) is a commonly used deep learning approach whereby a model designed for one problem can be reused to initiate a different but related task in deep learning. Due to the lack of annotated images and limited resources of computing power to train new models from scratch, transfer learning has become a popular method in deep learning, which can thus speed up the training process with fewer input data and improve the performance and generalizability of a deep learning model (10). Transfer learning with models trained using ImageNet has been extensively explored in medical imaging AI applications. The architectures of ResNet (11), Inception networks (12,13), and DenseNet (14) pretrained with ImageNet have been widely adopted and used in medical imaging applications for COVID-19 diagnosis at chest CT (15), classification of fibrotic lung disease (16), classification of skin cancer (17), and detection of acute intracranial hemorrhage (18).

Despite the high performance of many medical imaging models pretrained with ImageNet, previous works (19–21) have shown that pretrained models developed from medical source databases could achieve better performance than pretrained models from ImageNet. Successful transfer learning requires a reasonably large sample size, diversity of images, and similarity between the training and the target application images (22). In this study, we aim to create and evaluate a large-scale, diverse medical imaging dataset, RadImageNet, to generate pretrained convolutional neural networks (CNNs) trained solely from medical imaging to be used as the basis of transfer learning for medical imaging applications.

## Materials and Methods

### Study Patients

The institutional review boards waived the requirement for written informed consent for this retrospective, Health Insurance Portability and Accountability Act–compliant study, which evaluated de-identified data and involved no potential risk to patients. To avert any potential breach of confidentiality, no link between the patients, data provider, and data receiver was made available. A third party issued a certification of de-identified data transfer from the data provider to the data receiver. The RadImageNet dataset was collected between January 2005 and January 2020 from 131 872 patients at an outpatient radiology facility.

### Collection of RadImageNet Key Images

Each study was interpreted by a reading radiologist during daily clinical practice. A total of 20 board-certified, fellowship-trained radiologists participated in the original clinical interpretation. The radiologists had between 1 and 40 years of postfellowship experience at the time of clinical interpretation. As part of the interpretation of each study, the reading radiologist chose individual images representative of the pathologic finding or findings shown in each examination. The pathologic finding label was assigned to each of these “key images,” and a region of interest was created to localize the imaging findings. These original clinical interpretations were retrospectively extracted from the key images and provided the basis for the RadImageNet classes. The key images could be axial, sagittal, and/or coronal views and could include any sequence. The only requirement was that the image clearly represented substantial pathologic findings in the study.

### Normal Studies

To better investigate the characteristics of abnormal key images for model development, 8528 normal studies with 263 039 images were included. Normal studies were identified on the basis of a SQL query of the picture archiving and communication system database, whereby studies with a report containing “normal” or “unremarkable” in the impression were flagged. The findings and impressions of these studies were reviewed by a board-certified radiologist to confirm that the reports did indicate a completely normal study. Studies with abnormal but not clinically significant findings were excluded. All diagnostic sequences and images of these studies were included.

### Study Design

**Figure 1:**
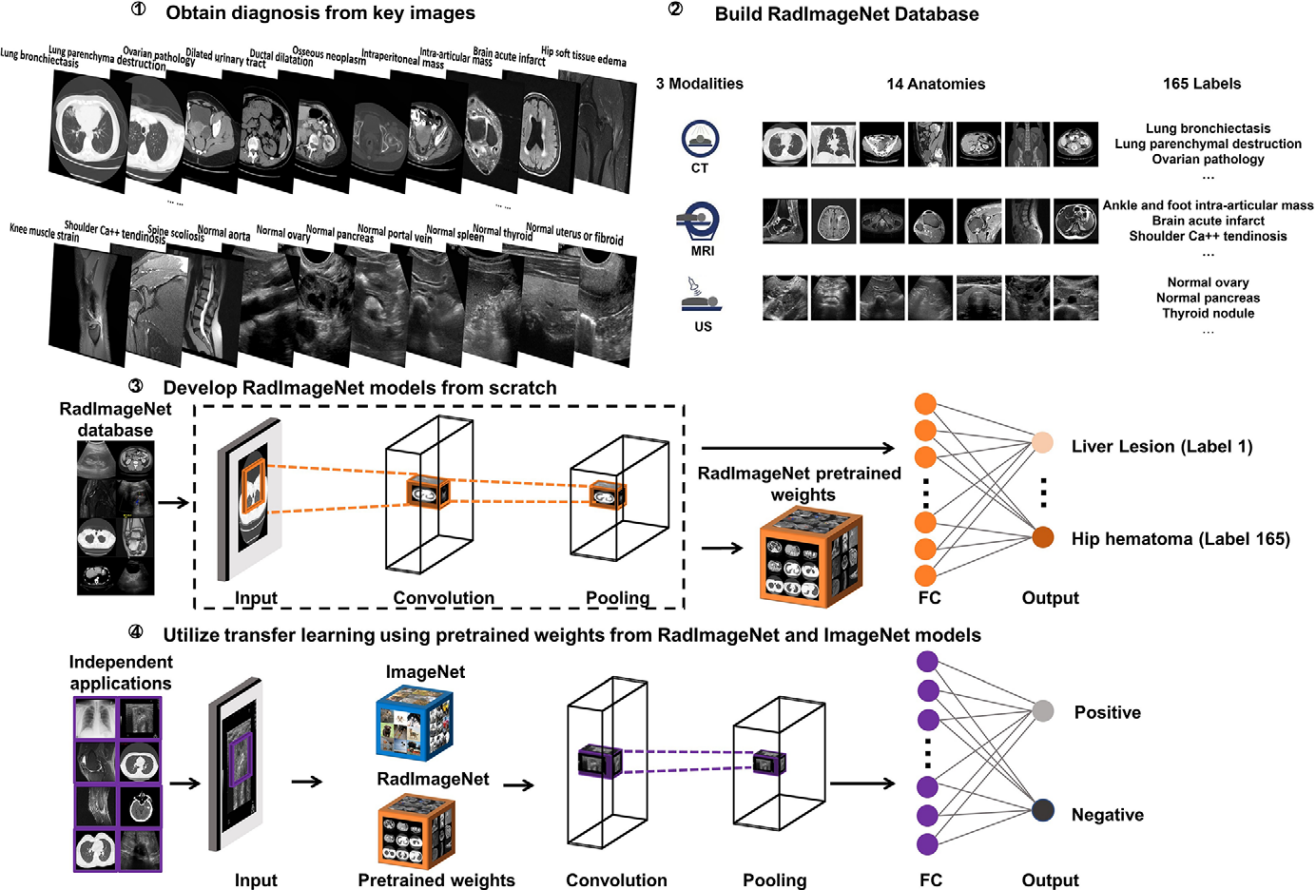
Curation of medical imaging database RadImageNet, development of pretrained convolutional neural networks over RadImageNet, and comparison of RadImageNet pretrained models and ImageNet pretrained models on multiple medical imaging applications.

This study was designed in four phases (Fig 1). First, key images and associated diagnoses were annotated by radiologists. Second, the images and diagnoses were further grouped by modalities, anatomic regions, and labels according to their imaging patterns to construct the medical imaging–only database RadImageNet. Third, four neural networks as pretrained models were trained from scratch based on RadImageNet. Finally, the pretrained models from RadImageNet and ImageNet were used and compared on eight medical imaging applications using area under the receiver operating characteristic curve (AUC) values and Dice scores if ground truth segmentation masks were available.

### RadImageNet Model Training

The same architectures of Inception-ResNet-v2 (13), ResNet50 (11), DenseNet121 (14), and InceptionV3 (12) networks were employed to train RadImageNet models from scratch by using randomly initiated weights as the starting point. The RadImageNet dataset was split into 75% training set, 10% validation set, and 15% test set. Images in the same patient were always included in the same set.

Rather than importing the weights from existing models, we randomly initiated the weights to develop the individual models. All images were resized to 224 × 224 pixels and used as the inputs of the neural networks. A global average pooling layer, a dropout layer at a rate of 0.5, and the output layer activated by the softmax function were added after the CNNs. The models returned a list of probabilities that the image corresponded to one of the 165 labels. The RadImageNet pretrained models and codes can be accessed at *https://github.com/BMEII-AI/RadImageNet*.

### Comparison of RadImageNet and ImageNet Pretrained Models

We applied RadImageNet and ImageNet pretrained models for transfer learning on eight external downstream applications. The eight tasks included the following: classification between malignant and benign thyroid nodules at US with 288 malignant images and 61 benign images (23); classification between malignant and benign breast lesions at US with 210 malignant images and 570 benign images (24); anterior cruciate ligament (ACL) and meniscus tear detection at MRI with 570 ACL tear images, 452 non-ACL tear images, 506 meniscal tear images, and 3695 nonmeniscal tear images (25); pneumonia detection on chest radiographs including 6012 pneumonia chest radiographs and 20 672 nonpneumonia images (26); differentiation of patients with COVID-19 from community-acquired pneumonia with 21 872 COVID-19 images and 36 894 community-acquired pneumonia images (27); classification between patients with and without COVID-19 at chest CT with 4190 COVID-19–positive and 4860 COVID-19–negative images (15); and hemorrhage detection at head CT with 107 933 hemorrhage images and 465 671 nonhemorrhage images (28). Detailed class distributions of the downstream datasets are reported in Tables E3 and E4 (supplement). The RadImageNet and ImageNet models were evaluated using receiver operating curve analysis. A total of 24 scenarios were simulated to fine-tune the models for each application. The four CNNs were trained with varied learning rates and different numbers of freezing layers. Unfreezing of all layers was conducted with learning rates of 0.001 and 0.0001, while the freezing of all layers and unfreezing of the top 10 layers were conducted with learning rates of 0.01 and 0.001. The average AUC and SD of these 24 settings were compared between RadImageNet and ImageNet pretrained models.

Each downstream application dataset was split into 75% training set, 10% validation set, and 15% test set. Images in one patient were always in the same set. Binary cross-entropy was selected as the loss function. The input images were downscaled to 256 × 256 pixels for the trade-off between accuracy and efficiency. A global average pooling layer, a dropout layer, and an output layer activated by the softmax function were introduced after the last layer of the pretrained models. Models were trained for 30 epochs. The models with the lowest validation loss in such epochs were saved for further evaluation and comparison on the test set. Experiments on thyroid, breast, ACL, and meniscus applications were conducted using fivefold cross-validation because of the small size of the dataset. The distribution and data split for each application are shown in Tables E3 and E4 (supplement).

### Gradient-weighted Class Activation Mapping

To understand the model interpretability, we used gradient-weighted class activation mapping (Grad-CAM) to visualize where the models make predictions in an image. Grad-CAM highlights the important regions in an image by using the gradients of the target layer that flows into the final convolutional layer to generate a localization map (29). For both RadImageNet and ImageNet models, the output layer was the target layer, whereas conv_7b_ac, conv5_block3_out, relu, and mixed10 were each selected as the final convolutional layer to generate the Grad-CAM for the Inception-ResNet-v2, ResNet50, DenseNet121, and InceptionV3 networks, respectively. Due to the varied recognition rates of CNN models for thyroid and breast classifications, a threshold of 180 was used to calculate Dice scores. This means that pixel values of a Grad-CAM that were greater than 180 for thyroid and breast US images were considered as predicted positives for further calculation.

### Statistical Analysis

The paired *t* test (30) was used to calculate the two-sided *P* value comparing Dice scores between the RadImageNet and ImageNet models. Each image in the thyroid and breast datasets is independent. The normality of the distribution of Dice scores generated by RadImageNet and ImageNet models was confirmed by the Shapiro test (31,32). The DeLong method (33) was used to evaluate the 95% CI of the AUC and to calculate the two-sided *P* value for the comparison of RadImageNet and ImageNet models. Statistical significance was defined as a *P* value less than .05. The statistics of AUC comparisons were computed in the pROC package (1.18.0) in R (version 4.1.3; R Foundation for Statistical Computing) (34). The Shapiro test and paired *t* test were performed in the statsmodels package (0.13.2) in Python 3.8.5.

## Results

### The RadImageNet Database

**Figure 2:**
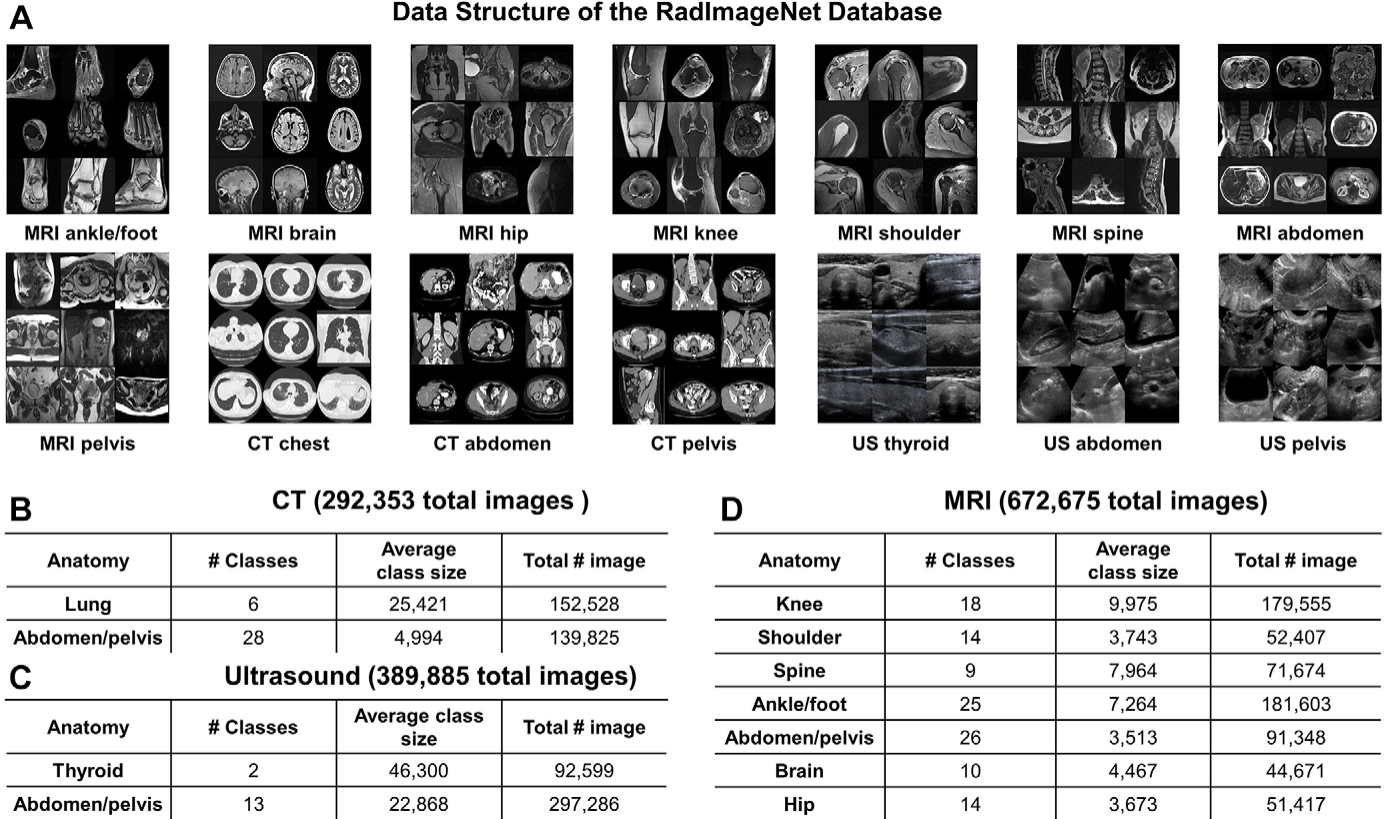
Representative images and data structure of the RadImageNet database. **(A)** Overview of RadImageNet modalities and anatomic regions. RadImageNet was constructed with CT, MRI, and US images, including CT of the chest, abdomen, and pelvis, MRI of the ankle, foot, knee, hip, shoulder, brain, spine, abdomen, and pelvis, and US of the abdomen, pelvis, and thyroid. These images represent the diversity and fundamental structure of the RadImageNet database. **(B–D)** The components of the RadImageNet database subdivided by modalities, anatomic regions, classes, and number of associated images within each anatomic region for **(B)** CT studies, **(C)** US studies, and **(D)** MRI studies.

The RadImageNet dataset includes 1.35 million annotated CT, MRI, and US images of musculoskeletal, neurologic, oncologic, gastrointestinal, endocrine, and pulmonary pathologic findings. For direct comparison with ImageNet (the initial size for the ImageNet challenge was 1.3 million images), we collected the most frequent modalities and anatomic regions on the same scale. The RadImageNet database used for comparison to ImageNet consists of three radiologic modalities, eleven anatomic regions, and 165 pathologic labels (Fig 2 and Table E1 [supplement]). The performance of the models on the test set is reported in Figure E1 and Table E2 (supplement).

### Performance on Small Datasets

**Figure 3:**
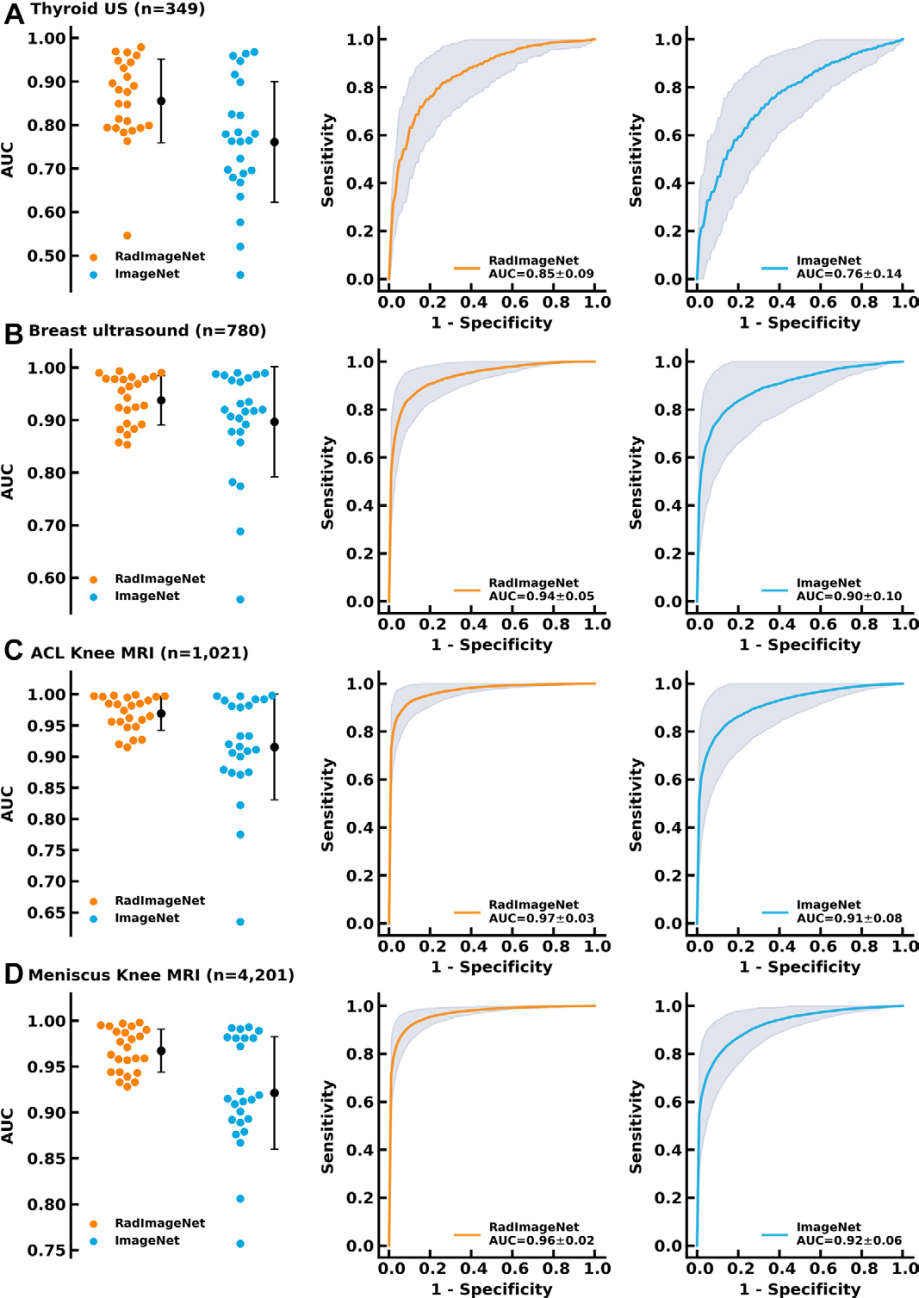
Performance of the RadImageNet pretrained models and ImageNet pretrained models on small datasets. The first column demonstrates swamp plots of 24 simulated experiments for RadImageNet and ImageNet models. The error bars represent the means and SDs on behalf of all area under the receiver operating characteristic curve (AUC) values. The second and third columns are receiver operating characteristic curves for the average of 24 simulations of RadImageNet models and ImageNet models, respectively. The shades indicate SDs obtained by averaging false-positive rates and true-positive rates from each experiment. The gains were calculated on the basis of the changes between RadImageNet and ImageNet models from ImageNet models. Two-sided *P* values were calculated by comparing the paired models between RadImageNet and ImageNet. AUC comparisons were evaluated by the DeLong test. **(A)** Thyroid US showed a 9.4% gain. **(B)** Breast US showed a 4.0% gain. **(C)** Anterior cruciate ligament (ACL) MRI showed a 4.8% gain. **(D)** Meniscus MRI showed a 4.5% gain.

The thyroid dataset contains 349 US images with radiologist-generated annotations collected from an open access thyroid image dataset (23). The breast dataset includes 780 breast US images (24) acquired for the detection of breast cancer. The knee MRI dataset consists of 1021 ACL tear and 4201 meniscal tear images (25). RadImageNet models demonstrated average AUCs of 0.85 ± 0.09 (SD) (*P* < .001), 0.94 ± 0.05 (*P* < .001), 0.97 ± 0.03 (*P* < .001), and 0.96 ± 0.02 (*P* < .001), compared with ImageNet models that showed values of 0.76 ± 0.14, 0.90 ± 0.10, 0.91 ± 0.08, and 0.92 ± 0.06 on the thyroid, breast, ACL tear, and meniscal tear datasets, respectively (Fig 3). The difference in AUCs between RadImageNet and ImageNet and the associated 95% CIs for the thyroid, breast, ACL tear, and meniscal tear datasets were 9.4% (2.6%, 16.2%), 4.0% (−0.6%, 8.7%), 4.8% (1.7%, 8.8%), and 4.5% (1.8%, 7.1%), respectively.

### Performance on Larger Datasets

**Figure 4:**
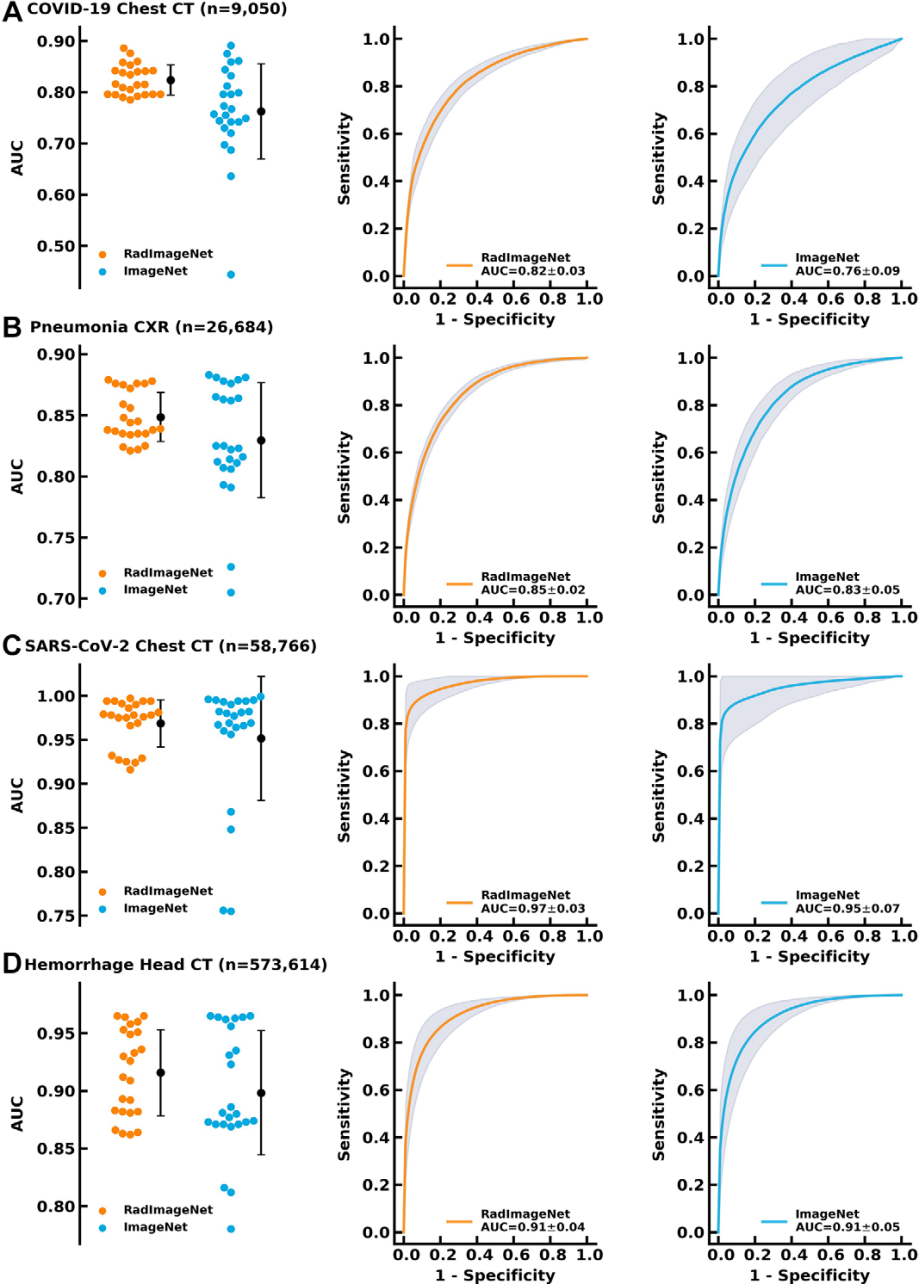
Performance of the RadImageNet pretrained models and ImageNet pretrained models on bigger datasets. The improvements from ImageNet are narrowed as compared with small datasets, but RadImageNet models are more consistent, showing smaller SDs among all simulations. **(A)** COVID-19 showed a 6.1% gain. **(B)** Pneumonia chest radiographs (CXR) showed a 1.9% gain. **(C)** SARS-CoV-2 CT showed a 1.7% gain. **(D)** Hemorrhage CT showed a 0.9% gain. AUC = area under the receiver operating characteristic curve.

The COVID-19 dataset consists of 9050 chest CT images in patients with and without COVID-19 pneumonia (15). The pneumonia dataset consists of 26 685 chest radiographs (26). The SARS-CoV-2 dataset consists of 58 766 chest CT images with and without SARS-CoV-2 pneumonia (27). The hemorrhage dataset consists of 573 614 head CT images with and without intracranial hemorrhage (28). On these four applications, the RadImageNet models demonstrated average AUCs of 0.82 ± 0.03 (*P* < .001), 0.85 ± 0.02 (*P* < .001), 0.97 ± 0.03 (*P* < .001), and 0.91 ± 0.04 (*P* < .001), outperforming ImageNet models, which showed mean AUCs of 0.76 ± 0.09, 0.83 ± 0.05, 0.95 ± 0.07, and 0.91 ± 0.05, respectively (Fig 4). The differences in AUCs between RadImageNet and ImageNet and the 95% CIs were indicated as 6.1% (2.2%, 10.0%), 1.9% (−0.2%, 4.0%), 1.7% (−1.4%, 4.7%), and 0.9% (−1.7%, 3.4%) for detection of COVID-19, pneumonia, SARS-CoV-2, and hemorrhage, respectively.

### Grad-CAM and Dice Scores

**Figure 5:**
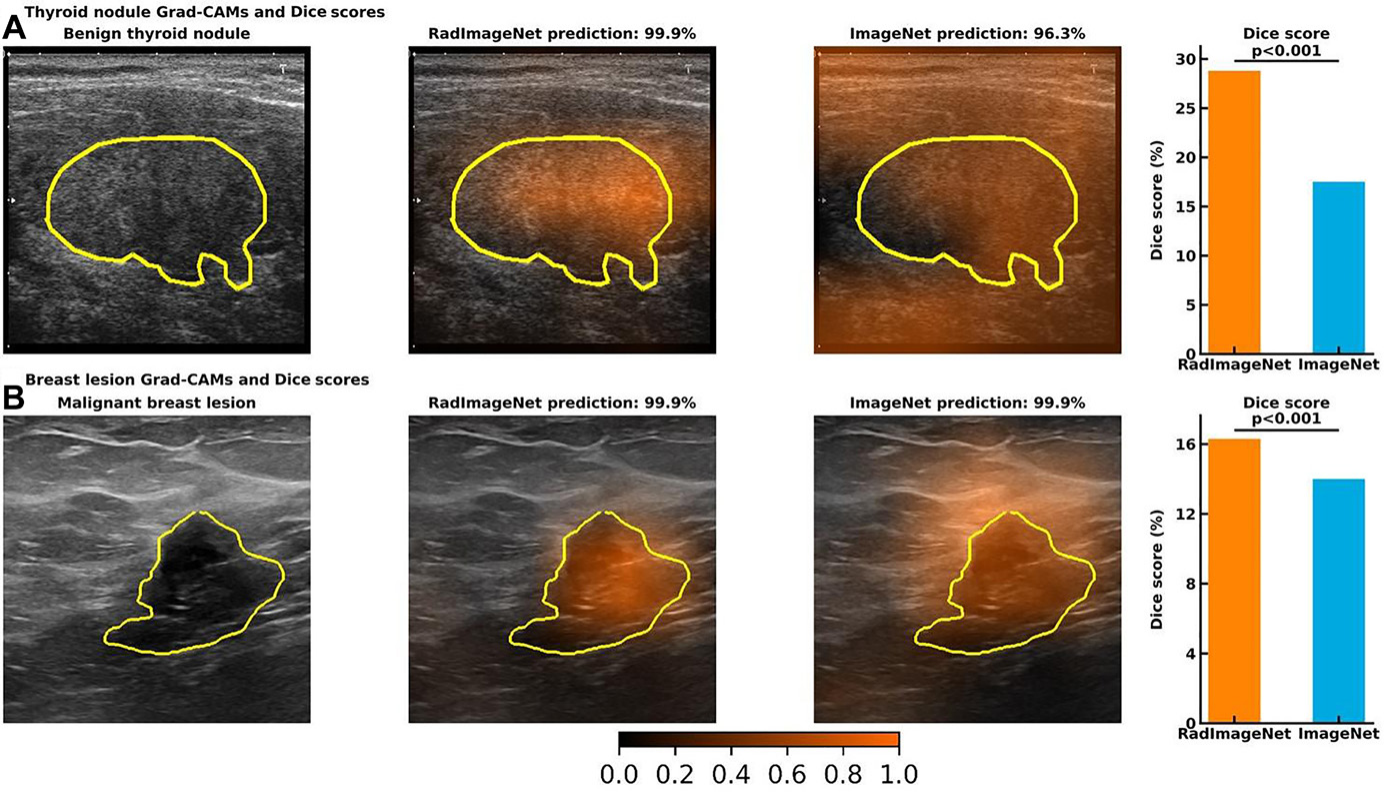
Visualizations of gradient-weighted class activation maps (Grad-CAMs) from accurately predicted images and Dice scores from the quantitative Grad-CAMs. The first column demonstrates the original image and the ground truth. The second and third columns show the Grad-CAMs from a RadImageNet model and an ImageNet model, respectively. The fourth column represents the average Dice score of 24 simulated RadImageNet and ImageNet models, with the error bar showing SDs. **(A)** A benign thyroid nodule. Both RadImageNet and ImageNet models accurately predicted the characteristics of this benign nodule, with the confidence of 99.9% and 96.3%, respectively. RadImageNet models achieved a Dice score of 28.8%, while ImageNet models had a Dice score of 17.5% (*P* < .001). **(B)** A malignant breast lesion. Both RadImageNet and ImageNet models accurately captured this malignant lesion, with confidence of 99.9% and 99.9%, respectively. RadImageNet models illustrated a 16.3% Dice score, outperforming ImageNet models, which showed a Dice score of 14.0% (*P* < .001). Two-sided *P* values for comparing the Dice scores were calculated by paired *t* test.

The thyroid and breast lesion US datasets contained graphical masks identifying the lesions. Dice score was used to evaluate the performance of the predicted CAM to the ground truth. Figure 5 shows CAM examples of RadImageNet models and ImageNet models. RadImageNet models achieved a Dice score of 0.29 for thyroid nodule detection, demonstrating a significant improvement of 64.6% from ImageNet models, which had a Dice score of 0.18 (*P* < .001). RadImageNet models illustrated a 0.16 Dice score for breast lesion detection, showing a significant gain of 16.4% from ImageNet models with a Dice score of 0.14 (*P* < .001).

## Discussion

In our study, the RadImageNet database contained only grayscale medical images, while natural world images use three red-green-blue channels. Pretraining on grayscale images can allow the training of more generalizable low-level filters in the initial layers of the network. The RadImageNet models demonstrated higher performance in imaging recognition and consistency over 24 simulated tuning scenarios regardless of the sample size of the applications. Within the 24 scenarios, unfreezing all layers consistently achieved the best performance as compared with unfreezing partial layers and training only fully connected layers. A smaller learning rate at 0.0001 would be suggested when training all trainable parameters to potentially better capture global optimal performance. If computational power and turnaround time allowed, Inception-ResNet-v2, ResNet50, and DenseNet121 achieved higher performance than Inception V3. However, the superiority of RadImageNet models is more evident on small datasets. For the four small datasets—thyroid US, breast US, and ACL and meniscus MRI—the RadImageNet models demonstrated a significant advantage over ImageNet models as demonstrated by a 9.4% (*P* < .001), 4.0% (*P* < .001), 4.8% (*P* < .001), and 4.5% (*P* < .001) AUC improvement, respectively. In addition, RadImageNet models are more stable. RadImageNet contained both the modality (US and MRI) and similar classes (normal thyroid and thyroid nodules, ACL injury and meniscus injury) to the target data, indicating that source data similarity can contribute to extraordinary performance with a small dataset. For the four relatively larger datasets—pneumonia detection at chest radiography (26 684 images), COVID-19 CT (9050 images), SARS-CoV-2 CT (58 766 images), and intracranial hemorrhage detection CT (573 614 images)—the RadImageNet models also illustrated improvements of AUC by 1.9% (*P* < .001), 6.1% (*P* < .001), 1.7% (*P* < .001), and 0.9% (*P* < .001), respectively. RadImageNet contained both the modality (CT and MRI) and similar classes (infections on chest CT images and brain injuries on brain MRI studies) but with no radiography data involved. This indicated that even though the similarity to the target data was not high, the diversity of sourcing data and the larger sample size of the target data could compensate for model performance.

Moreover, RadImageNet models showed better generalizability. For the three applications (breast US, pneumonia chest radiography, and hemorrhage CT) in which the classes, anatomic regions, or modalities were not all included in the RadImageNet database, RadImageNet models demonstrated comparable performance by showing improvements of 4.0%, 1.9%, and 0.9%, respectively. These improvements confirmed that RadImageNet models are applicable to medical datasets regardless of modalities, anatomic regions, and classes.

In addition to the superiority of RadImageNet models in classification tasks, the interpretation of the RadImageNet models results more closely matched ground truth as compared with the ImageNet models. The quantification of the class activation maps for thyroid and breast images showed that RadImageNet models are more explainable and more consistent than ImageNet models, demonstrating improvements in Dice scores relative to ImageNet models of 64.6% and 16.4%, respectively. These results further confirmed that the similarity of the features learned from the RadImageNet database contributed to a higher recognition rate with better interpretability of the models (Fig 5).

Our proposed RadImageNet models did have limitations. First, the evaluation of pathologic features on a single image by radiologists at a single radiology facility and RadImageNet models did not mimic clinical diagnostic workflow, likely resulting in the similar, but poor, diagnostic performance by both. Second, the images presented may have contained multiple pathologic features, but we only used one label. Moreover, the region of interest placed on the primary pathologic finding by the reading radiologist was not used in this study. Therefore, it is possible the radiologists or RadImageNet models correctly matched a pathologic finding shown on the image but not the one that corresponded to the primary label. Third, the RadImageNet models used reduced-resolution images in algorithm development because of processing limitations. These lower-resolution images may obscure small areas of pathologic findings. Fourth, the current 165 categories were grouped on the basis of the *International Classification of Diseases, Tenth Revision,* and imaging characteristics, thus the RadImageNet models could not be diagnostic models to assist human experts. Finally, the number of classes in the limited RadImageNet dataset used for comparison to ImageNet was less than the number in ImageNet.

In conclusion, RadImageNet pretrained models could serve as a better starting point for transfer learning approaches in medical imaging analysis. In future studies, higher-spatial-resolution images could result in higher performance for recognition of smaller foci of pathologic features. Other imaging modalities such as radiography and PET should be included. The number of classes of pathologic findings in RadImageNet can be further expanded. Moreover, performance could be improved by introducing the regions of interest, as defined by radiologists, to highlight pathologic appearance in the images, as well as by providing additional sequences and/or adjacent images for the development of diagnostic models. Finally, more fine-tuning the pretrained models and comparing them with the standard pretrained models will be further analyzed.

## References

[1] Deng J , Dong W , Socher R , Li LJ , Li K , Fei-Fei L . ImageNet: A large-scale hierarchical image database . In: 2009 IEEE Conference on Computer Vision and Pattern Recognition , Miami, FL , June 20–25, 2009 . Piscataway, NJ : IEEE , 2009 ; 248 – 255 .

[2] Russakovsky O , Deng J , Su H , et al . ImageNet large scale visual recognition challenge . Int J Comput Vis 2015 ; 115 ( 3 ): 211 – 252 .

[3] Langlotz CP , Allen B , Erickson BJ , et al . A roadmap for foundational research on artificial intelligence in medical imaging: from the 2018 NIH/RSNA/ACR/The Academy Workshop . Radiology 2019 ; 291 ( 3 ): 781 – 791 . 30990384 10.1148/radiol.2019190613 PMC6542624

[4] Bycroft C , Freeman C , Petkova D , et al . The UK Biobank resource with deep phenotyping and genomic data . Nature 2018 ; 562 ( 7726 ): 203 – 209 . 30305743 10.1038/s41586-018-0579-z PMC6786975

[5] Sudlow C , Gallacher J , Allen N , et al . UK Biobank: an open access resource for identifying the causes of a wide range of complex diseases of middle and old age . PLoS Med 2015 ; 12 ( 3 ): e1001779 . 25826379 10.1371/journal.pmed.1001779 PMC4380465

[6] Clark K , Vendt B , Smith K , et al . The Cancer Imaging Archive (TCIA): maintaining and operating a public information repository . J Digit Imaging 2013 ; 26 ( 6 ): 1045 – 1057 . 23884657 10.1007/s10278-013-9622-7 PMC3824915

[7] National Lung Screening Trial Research Team ; Aberle DR , Adams AM , et al . Reduced lung-cancer mortality with low-dose computed tomographic screening . N Engl J Med 2011 ; 365 ( 5 ): 395 – 409 . 21714641 10.1056/NEJMoa1102873 PMC4356534

[8] Wang X , Peng Y , Lu L , Lu Z , Bagheri M , Summers RM . ChestX-Ray8: hospital-scale chest x-ray database and benchmarks on weakly-supervised classification and localization of common thorax diseases . In: 2017 IEEE Conference on Computer Vision and Pattern Recognition (CVPR) , Honolulu, HI , July 21–26, 2017 . Piscataway, NJ : IEEE , 2017 ; 3462 – 3471 .

[9] Pan SJ , Yang Q . A survey on transfer learning . IEEE Trans Knowl Data Eng 2010 ; 22 ( 10 ): 1345 – 1359 .

[10] Agrawal P , Girshick R , Malik J . Analyzing the performance of multilayer neural networks for object recognition . In: Fleet D , Pajdla T , Schiele B , Tuytelaars T , eds. Computer Vision – ECCV 2014. ECCV 2014. Lecture Notes in Computer Science, vol 8695 . Cham, Switzerland : Springer , 2014 ; 329 – 344 .

[11] He K , Zhang X , Ren S , Sun J . Deep residual learning for image recognition . In: 2016 IEEE Conference on Computer Vision and Pattern Recognition (CVPR) , Las Vegas, NV , June 27–30, 2016 . Piscataway, NJ : IEEE , 2016 ; 770 – 778 .

[12] Szegedy C , Vanhoucke V , Ioffe S , Shlens J , Wojna Z . Rethinking the inception architecture for computer vision . In: 2016 IEEE Conference on Computer Vision and Pattern Recognition (CVPR) , Las Vegas, NV , June 27–30, 2016 . Piscataway, NJ : IEEE , 2016 ; 2818 – 2826 .

[13] Szegedy C , Ioffe S , Vanhoucke V , Alemi A . Inception-v4, Inception-ResNet and the impact of residual connections on learning . Proc Conf AAAI Artif Intell 2017 ; 31 ( 1 ).

[14] Huang G , Liu Z , van der Maaten L , Weinberger KQ . Densely connected convolutional networks . In: 2017 IEEE Conference on Computer Vision and Pattern Recognition (CVPR) , Honolulu, HI , July 21–26, 2017 . Piscataway, NJ : IEEE , 2017 ; 2261 – 2269 .

[15] Mei X , Lee HC , Diao KY , et al . Artificial intelligence-enabled rapid diagnosis of patients with COVID-19 . Nat Med 2020 ; 26 ( 8 ): 1224 – 1228 . 32427924 10.1038/s41591-020-0931-3 PMC7446729

[16] Walsh SLF , Calandriello L , Silva M , Sverzellati N . Deep learning for classifying fibrotic lung disease on high-resolution computed tomography: a case-cohort study . Lancet Respir Med 2018 ; 6 ( 11 ): 837 – 845 . 30232049 10.1016/S2213-2600(18)30286-8

[17] Esteva A , Kuprel B , Novoa RA , et al . Dermatologist-level classification of skin cancer with deep neural networks . Nature 2017 ; 542 ( 7639 ): 115 – 118 . [Published correction appears in Nature 2017;546(7660):686.] 28117445 10.1038/nature21056 PMC8382232

[18] Lee H , Yune S , Mansouri M , et al . An explainable deep-learning algorithm for the detection of acute intracranial haemorrhage from small datasets . Nat Biomed Eng 2019 ; 3 ( 3 ): 173 – 182 . 30948806 10.1038/s41551-018-0324-9

[19] Xie Y , Richmond D . Pre-training on grayscale imagenet improves medical image classification . In: Leal-Taixé L , Roth S , eds. Computer Vision – ECCV 2018 Workshops. ECCV 2018. Lecture Notes in Computer Science, vol 11134 . Cham, Switzerland : Springer , 2019 ; 476 – 484 .

[20] Parakh A , Lee H , Lee JH , Eisner BH , Sahani DV , Do S . Urinary stone detection on ct images using deep convolutional neural networks: evaluation of model performance and generalization . Radiol Artif Intell 2019 ; 1 ( 4 ): e180066 . 33937795 10.1148/ryai.2019180066 PMC8017404

[21] Ghesu FC , Georgescu B , Mansoor A , et al . Self-supervised Learning from 100 Million Medical Images . arXiv:2201.01283 [preprint] https://arxiv.org/abs/2201.01283. Posted January 4, 2022. Accessed February 14, 2022 .

[22] Cheplygina V , de Bruijne M , Pluim JPW . Not-so-supervised: A survey of semi-supervised, multi-instance, and transfer learning in medical image analysis . Med Image Anal 2019 ; 54 : 280 – 296 . 30959445 10.1016/j.media.2019.03.009

[23] Pedraza L, Vargas C, Narváez F, Durán O, Muñoz E, Romero E. An open access thyroid ultrasound image database. In: Romero E, Lepore N, eds. Proceedings of SPIE: 10th International Symposium on Medical Information Processing and Analysis. Vol 9287. Bellingham, Wash: International Society for Optics and Photonics, 2015; 92870W.

[24] Al-Dhabyani W , Gomaa M , Khaled H , Fahmy A . Dataset of breast ultrasound images . Data Brief 2019 ; 28 : 104863 . 31867417 10.1016/j.dib.2019.104863 PMC6906728

[25] Bien N , Rajpurkar P , Ball RL , et al . Deep-learning-assisted diagnosis for knee magnetic resonance imaging: Development and retrospective validation of MRNet . PLoS Med 2018 ; 15 ( 11 ): e1002699 . 30481176 10.1371/journal.pmed.1002699 PMC6258509

[26] Shih G , Wu CC , Halabi SS , et al . Augmenting the national institutes of health chest radiograph dataset with expert annotations of possible pneumonia . Radiol Artif Intell 2019 ; 1 ( 1 ): e180041 . 33937785 10.1148/ryai.2019180041 PMC8017407

[27] Zhang K , Liu X , Shen J , et al . Clinically applicable AI system for accurate diagnosis, quantitative measurements, and prognosis of COVID-19 pneumonia using computed tomography . Cell 2020 ; 181 ( 6 ): 1423 – 1433.e11 . 32416069 10.1016/j.cell.2020.04.045 PMC7196900

[28] Flanders AE , Prevedello LM , Shih G , et al . Construction of a machine learning dataset through collaboration: The RSNA 2019 Brain CT Hemorrhage Challenge . Radiol Artif Intell 2020 ; 2 ( 3 ): e190211 [Published correction appears in Radiol Artif Intell 2020;2(4):e209002.] . 33937827 10.1148/ryai.2020190211 PMC8082297

[29] Selvaraju RR , Cogswell M , Das A , Vedantam R , Parikh D , Batra D . Grad-CAM: visual explanations from deep networks via gradient-based localization . Int J Comput Vis 2020 ; 128 ( 2 ): 336 – 359 .

[30] David HA , Gunnink JL . The paired t test under artificial pairing . Am Stat 1997 ; 51 ( 1 ): 9 – 12 .

[31] Shapiro SS , Wilk MB . An analysis of variance test for normality (complete samples) . Biometrika 1965 ; 52 ( 3/4 ): 591 – 611 .

[32] Mohd Razali N , Yap BW . Power comparisons of Shapiro-Wilk, Kolmogorov-Smirnov, Lilliefors and Anderson-Darling tests . J Stat Model Anal 2011 ; 2 ( 1 ): 21 – 33 . https://www.nrc.gov/docs/ML1714/ML17143A100.pdf .

[33] DeLong ER , DeLong DM , Clarke-Pearson DL . Comparing the areas under two or more correlated receiver operating characteristic curves: a nonparametric approach . Biometrics 1988 ; 44 ( 3 ): 837 – 845 . 3203132

[34] Robin X , Turck N , Hainard A , et al . pROC: an open-source package for R and S+ to analyze and compare ROC curves . BMC Bioinformatics 2011 ; 12 ( 1 ): 77 . 21414208 10.1186/1471-2105-12-77 PMC3068975

[35] Zatz LM . Basic principles of computed tomography scanning . In: Newton TH , Potts DG , eds. Technical Aspects of Computed Tomography . St Louis, Mo : Mosby , 1981 ; 3853 – 3876 .

[36] Seeram E . Computed Tomography-E-Book: Physical Principles, Clinical Applications, and Quality Control . St Louis, Mo : Elsevier Health Sciences , 2015 .

